# Phytochemistry and bioactivity of aromatic and medicinal plants from the genus *Agastache* (*Lamiaceae*)

**DOI:** 10.1007/s11101-014-9349-1

**Published:** 2014-04-03

**Authors:** Sylwia Zielińska, Adam Matkowski

**Affiliations:** Department of Pharmaceutical Biology and Botany, Medical University of Wroclaw, Borowska 211, 50-556 Wroclaw, Poland

**Keywords:** Essential oil, Estragole, Giant hyssop, Phenylpropanoids, Tilianin

## Abstract

*Agastache* is a small genus of *Lamiaceae*, comprising 22 species of perennial aromatic medicinal herbs. In this article, we review recent advances in phytochemical, pharmacological, biotechnological and molecular research on *Agastache*. The phytochemical profile of all *Agastache* species studied to date is generally similar, consisted of two main metabolic classes—phenylpropanoids and terpenoids. In the relatively variable essential oils, most populations of different *Agastache* species contain over 50 % of a phenylallyl compound—estragole. Also, other volatile compounds (methyleugenol, pulegone, menthone, isomenthone and spathulenol) were reported in various proportions. Major non-volatile metabolites belong to phenolic compounds, such as caffeic acid derivatives, especially rosmarinic acid as well as several flavones and flavone glycosides like acacetin, tilianin, agastachoside, and a rare dimeric malonyl flavone (agastachin). Two unique lignans—agastenol and agastinol—were also isolated. Terpenoids include triterpenoids of oleanane-type (maslinic acid, oleanolic acid and β-amyrin), ursane-type (ursolic acid, corosolic acid and α-amyrin), and typical plant sterols, as well as abietane-type oxidized diterpenes (e.g., agastaquinone, agastol, and others). The bioactivity of various extracts or individual compounds in vitro and in vivo include antimicrobial, antiviral and anti-mutagenic activity, cytotoxic activity to cancer cell lines, and anti-nociceptive, anti-inflammatory, anti-atherogenic, antioxidant as well as biocidal activity to several foodstuff pests. Biotechnological and molecular studies have focused on in vitro propagation and enhancing the biosynthesis of bioactive metabolites in cell or organ cultures, as well as on the expression of genes involved in phenolic biosynthesis.

## Introduction

Throughout the world, hundreds of *Lamiaceae* (Mint family) species are used as medicinal and aromatic plants. Some of them are among the most popular spices and herbs, like basil, peppermint, sage, and many others. Here, we would like to portray one genus—*Agastache* Clayt. ex Gronov.—that has similar properties but which is not as commonly recognized. Plants from this genus are known under the vernacular name ‘giant hyssop’. Some of these plants are utilized as a source of essential oil, herbal drugs, spice, nectariferous plants in beekeeping, or as ornamentals. Their ornamental use is actually the most common, making giant hyssops one of a few examples in the mint family where decorative value appears to overshadow its potential as a medicinal plant. Almost 16 years have passed since the last (and only) systematic review of the *Agastache* genus was published (Fuentes-Granados et al. 1998). Since then, significant progress in biological investigations has been made. Apart from the morphological, cytogenetical, taxonomic, horticultural and biochemical research that has been previously described, several important biotechnological and molecular studies of *Agastache* species have appeared in the meantime.

As a result of increasing interest in ethnic and traditional phytotherapeutics, many new studies have been undertaken to examine the pharmacological properties of these herbs, including a few *Agastache* species. So far, only a few species of the genus *Agastache* have been fairly represented in the phytochemical and pharmacological literature. Even so, the available data sufficiently support the prospect of increasing use of *Agastache* *spp.* and their constituents in herbal therapy.

In the present review, we discuss recent advances in the phytochemistry, bioactivity, molecular biology and biotechnology of *Agastache*, with an emphasis on the following species: *A. foeniculum*, *A. mexicana*, *A. rugosa*, *A. scrophulariifolia* and *A. urticifolia*. However, informations about other species are also considered upon their availability in the literature.

For a comprehensive literature overview, we analyzed the published phytochemical and pharmacological data available through several search engines, such as ^®^SciFinder, ISI ^®^Web of Science, ^®^Scopus and ^®^GoogleScholar, using ‘*Agastache*’ as the search keyword. We disregarded publications pertaining to agronomy, plant pathology, ecology and other unrelated topics (unless any phytochemical or pharmacological data were provided in them). In a few cases, we found publications using bibliographical data from the reference lists of newer papers to verify the cited information. The major portion of the retrieved citations came from years following the previous review paper of 1998 (Fuentes-Granados et al. 1998).

## Botanical description

The genus *Agastache* belongs to the *Nepetoideae*—a subfamily of the *Lamiaceae* (Cantino et al. 1992). The species of *Agastache* can be separated into two sections: *Brittonastrum* and *Agastache* (Lint and Epling 1945; Sanders 1987).

Plants from *Agastache* genus are perennial herbs, reaching one meter or more in height. The stems can be simple or branched, erect or slightly creeping, and with an occasionally woody stem base. Their morphology is typical for *Lamiaceae*, with opposite petiolate leaves, a four-angled stem, numerous trichomes and labiate flowers with pink, purple, white, yellowish or orange corolla. The base chromosome number is 9. *Agastache* species are native to North America, but one species occurs naturally in East Asia (*A. rugosa*). Several species are cultivated as ornamentals, with numerous spectacular blooming cultivars. According to the current listing by the taxonomical Internet database lead by the Royal Botanical Gardens at Kew and the Missouri Botanical Garden (www.theplantlist.org—accessed 31 December 2013), the genus *Agastache* encompasses the following 29 accepted taxons:

- *A.* *aurantiaca* (A.Gray) Lint & Epling
- *A.* *breviflora* (A.Gray) Epling
- *A.* *cana* (Hook.) Wooton & Standl.
- *A.* *coccinea* (Greene) Lint & Epling
- *A.* *cusickii* (Greenm.) A.Heller
- *A.* *eplingiana* R.W.Sanders
- *A.* *foeniculum* (Pursh) Kuntze
- *A.* *mearnsii* Wooton & Standl.
- *A.* *mexicana* (Kunth) Lint & Epling
- *A.* *mexicana* subsp. *mexicana* (an infraspecific taxon)
- *A.* *micrantha* (A.Gray) Wooton & Standl.
- *A.* *nepetoides* (L.) Kuntze
- *A.* *occidentalis* (Piper) A.Heller
- *A.* *pallida* (Lindl.) Cory
- *A.* *pallida* var. *pallida* (an infraspecific taxon)
- *A.* *pallidiflora* (A.Heller) Rydb.
- *A.* *pallidiflora* var. *greenei* (Briq.) R.W.Sanders
- *A.* *pallidiflora* var. *harvardii* (A.Gray) R.W.Sanders
- *A.* *pallidiflora* subsp. *neomexicana* (Briq.) Lint & Epling
- *A.* *palmeri* (B.L.Rob.) Standl.
- *A.* *palmeri* var. *breviflora* (Regel) R.W.Sanders
- *A.* *parvifolia* Eastw.
- *A.* *pringlei* (Briq.) Lint & Epling
- *A.* *pringlei* var. *verticillata* (Wooton & Standl.) R.W.Sanders
- *A.* *rugosa* (Fisch. & C.A.Mey.) Kuntze
- *A.* *rupestris* (Greene) Standl.
- *A.* *scrophulariifolia* (Willd.) Kuntze
- *A.* *urticifolia* (Benth.) Kuntze
- *A.* *wrightii* (Greenm.) Wooton & Standl.

However, the number of *Agastache* species recognized has not been constant, and several of them have moved taxonomical positions throughout the years (Lint and Epling 1945; Vogelmann 1985; Sanders 1987; Fuentes-Granados et al. 1998; RBG Kew—the plant list web-based resource, accessed 31 December 2013). In 1945, 22 taxa of *Agastache* were reported (Lint and Epling 1945), divided into two sections: *Brittonastrum* and *Chiastandra* (synonymous with currently recognized section *Agastache*). *Brittonastrum* was described as native to the southwestern United States and Mexico, while *Chiastandra* was described in relation to the northern part of the United States, Canada and East Asia (Lint and Epling 1945). Later, one more species was recognized (*A. eplingiana*) in an extensive taxonomic study of *Brittonastrum* (Sanders 1987), two more were separated from those already established (*A. pallida* and *A. mearnsii*), and one species was divided into two subspecies, with two or three varieties (*A. pallidiflora*: *A. p.* var. *greenei* and *A. p.* var. *havardii*).

In 1998, Fuentes-Granados et al. enumerated 22 species of the genus *Agastache* which had been previously described (Lint and Epling 1945; Vogelmann 1985; Sanders 1987). The placing of a species in either of the two sections is based chiefly on differences in stamen length and arrangement. Although the main criterion of the subgeneric partition seems to be clear-cut, the genetic relationships between the species indicate more complexity (Lint and Epling 1945). Furthermore, morphological similarities between species of the same section do not necessarily indicate close genetic affinity (Table 1). Analysis of isoenzyme profiles and multivariate morphological classification have demonstrated that both morphological features and genetic distances between species of both sections are not consistently greater than within each section (Vogelmann 1985; Sanders 1987). *A. rugosa* appeared to be more similar to the eastern North-American populations than to the western North-American populations of sect. *Agastache* (Vogelmann and Gastony 1987). Moreover, there is less intraspecific variation in the *Agastache* section than in *Brittonastrum* (Lint and Epling 1945).

**Table 1:** Comparison of selected botanical traits of six *Agastache* species

Species	Common name	Distribution	Corolla color	Height [cm]	References
A. foeniculum* (Pursh) O. Kuntze Britton	Blue (giant) hyssop, anise hyssop	Northern Great Plains *** Western Great Lakes***	Blue	Less than 100	Lint and Epling (1945), Ayers and Widrlechner (1994)
A. nepetoides* (L.) O. Kuntze	Yellow/catnip (giant) hyssop	Southern New England Southern Great Lakes*** Ohio River Basin Ozarks	Greenish yellow	100 or more	Lint and Epling (1945), Ayers and Widrlechner (1994)
A. rugosa* (Fisch. & C.A. Mey.) O. Kuntze	Wrinkled (giant) hyssop, Korean mint	Korea, East China Japan, Manchuria and Russian Far East	Purplish blue	100 or more	Lint and Epling (1945), Ayers and Widrlechner (1994)
A. scrophulariifolia* (Wilde) O. Kuntze	Purple (giant) hyssop, figwort (giant) hyssop, prairie hyssop	Southern New England Southern to Western South Carolina Western to Northern Missouri Southern Minnesota	Pale pink to purple	Up to 210	Lint and Epling (1945), Ayers and Widrlechner (1994), Corrigan (2002)
A. urticifolia* (Benth.)	Nettle-leaf (giant) hyssop, horse nettle	Sierra Nevada Eastern Cascades Great Basin Northern Rockies***	Bright purple and pink	100–200 or more	Lint and Epling (1945), Ayers and Widrlechner (1994), Manning and Padgett (1991)
A. mexicana** (Kunth) Lint & Epling	Mexican (giant) hyssop, toronjil morado/rojo/colorado, nahuatl, tepehua	Cuijingo and Ozumba, Mexico	Purplish red to red	50–150	Sanders (1987), Hersch-Martinez (1997), Ibarra-Alvarado et al. (2010)

* Sect. *Agastache*

** Sect. *Brittonastrum*

*** USA/Canada

The morphology of leaf laminas as well as stem-types and inflorescence were described in detail in Fuentes-Granados et al.’s review (1998) and some earlier papers (Lint and Epling 1945; Vogelmann 1985; Sanders 1987). Generally, the leaves of plants from the *Agastache* section are longer (up to 15 cm) than those of *Brittonastrum* (2–6 cm). Plants from the *Agastache* section have ovate laminas with a crenate-serrate leaf margin, whereas those of *Brittonastrum* are more diverse. In the latter section, the basic leaf form is cordate-triangular, but juvenile laminas are ovate-to-cordate and mature—cordate, ovate, narrowly ovate or oblong-linear. Leaf margins are usually crenate, sometimes entire (Sanders 1987). The stems of plants from the *Agastache* section are simple or else branched with dense spicate inflorescences formed at terminal apices (Lint and Epling 1945). The inflorescence of *Brittonastrum* plants is basically an elongate thyrse. However, in various species and environmental conditions, it can be either continuous and spike-like-to-brush-like or else discontinuous and moniliform-to-loosely ramified, with a lower cymose clusters often remote from the upper (Sanders 1987). However, the latter feature can also appear in species from the *Agastache* section (Fuentes-Granados et al. 1998).

The morphology of *Agastache* and *Brittonastrum* flowers is also different (Sanders 1987). A typical *Agastache* section corolla is asymmetrically and narrowly funnel-formed, and slightly two-lipped. Two adaxial lobes are fused for about two-thirds of their length into a shallowly concave upper lip. Two lateral lobes are much exceeded by the upper lip. Four stamens are exserted from the tube and included under the greatly exceeding upper corolla lip. The dorsal pair of stamens is longer (didynamous). In *Brittonastrum*, the corolla mouth is oblique with small lobes (less than one-quarter of the entire corolla length). The lateral lobes are fused more to the upper lip than to the median lobe. Stamens are included under or shortly exerted beyond the upper corolla lip.

From the several species considered here (*A. rugosa*, *A. foeniculum*, *A. urticifolia*, *A. scrophulariifolia*, *A. mexicana* and *A. nepetoides*), only *A. mexicana* is a member of the *Brittonastrum* section. The morphological and chemical investigations of *A. mexicana* suggested a new, white flowering taxon named *A. mexicana* subsp*. xolocotziana* Bye, Linares & Ramamoorthy (Bye et al. 1987). Hence, *A. mexicana* has been placed into the nomotypical subspecies *A. mexicana* subsp*. mexicana*. See also the ‘phytochemistry’ section below for details on the chemical differences between these two subspecies.

- **Synonyms of the five species covered by the present review** (theplantlist.org)
- sect. *Agastache*

### ***Agastache foeniculum*(Pursh) O. Kuntze Britton**

(*Agastache anethiodora* (Nutt.) Britton & A.Br.*, Agastache foeniculum* f. *bernardii* B. Boivin, *Agastache foeniculum* f. *candicans* B. Boivin*, Hyptis marathrosma* (Spreng.) Benth., *Hyssopus anethiodorus* Nutt., *Hyssopus anisatus* Nutt., *Hyssopus discolor* Desf., *Hyssopus foeniculum* (Pursh) Spreng., *Lophanthus anisatus* (Nutt.) Benth., *Lophanthus foeniculum* (Pursh) E.Mey., *Perilla marathrosma* Spreng., *Stachys foeniculum* Pursh, *Vleckia albescens* Raf., *Vleckia anethiodora* (Nutt.) Greene, *Vleckia anisata* (Nutt.) Raf., *Vleckia bracteata* Raf., *Vleckia bracteosa* Raf., *Vleckia discolor* Raf., *Vleckia foeniculum* (Pursh) MacMill., *Vleckia incarnate* Raf.)

### ***Agastache rugosa*(Fisch. & C.A. Mey.) O. Kuntze**

*(Agastache formosana* (Hayata) Hayata ex Makino & Nemoto, *Agastache rugosa* f*. alba* Y.N.Lee, *Cedronella japonica* Hassk., *Elsholtzia monostachys* H.Lév. & Vaniot, *Lophanthus argyi* H.Lév., *Lophanthus formosanus* Hayata, *Lophanthus rugosus* Fisch. & C.A.Mey.)

### ***Agastache scrophulariifolia*(Wilde) O. Kuntze**

*(Agastache scrophulariifolia* var. *mollis* (Fernald) A.Heller, *Hyssopus catariifolius* Benth., *Hyssopus scrophulariifolius* Willd., *Lophanthus scrophulariifolius* (Willd.) Benth., *Lophanthus scrophulariifolius* var. *mollis* Fernald, *Vleckia cordifolia* Raf., *Vleckia scrophularifolia* (Willd.) Raf.)

### ***Agastache urticifolia*(Benth.) Kuntze**

*(Agastache glaucifolia* A.Heller, *Agastache urticifolia* var. *glaucifolia* (A.Heller) Cronquist, *Lophanthus urticifolius* Benth., *Vleckia urticifolia* (Benth.) Raf.).

- sect. *Brittonastrum*

### ***Agastache mexicana*(Kunth) Lint & Epling**

*(Brittonastrum mexicanum* (Kunth) Briq., *Cedronella mexicana* (Kunth) Benth., *Dracocephalum mexicanum* Kunth, *Dekinia coccinea* Martens & Galeotti, *Gardoquia betonicoides* Lindley).

## Phytochemistry

*Agastache* species—typically for *Lamiaceae*—are abundant in phenylpropanoid and terpenoid specialized metabolites. The first group includes flavonoids, free phenolic acids and depsides as well as lignans. The second major group—terpenoids are contained in volatile fractions as well as in various organs as non-volatiles. Most of the published studies focus on essential oil analysis. There are also numerous papers reporting the isolation and elucidation of the structure of various phytochemicals.

### Overview of extraction, analysis and purification methods

Determination of volatile constituents:

- Essential oil hydrodistillation using Clevenger apparatus or pharmacopoeia distillation apparatus (Charles et al. 1991; Mazza and Kiehn 1992; Svoboda et al. 1995; Dung et al. 1996; Tirillini et al. 1997; Dapkevicius et al. 1998; Kim et al. 2001a; Maruyama et al. 2002; Shin and Kang 2003; Omidbaigi and Sefidkon 2003, 2004; Estrada-Reyes et al. 2004; Mallavarapu et al. 2004; Shin and Pyun 2004; Bruni et al. 2007; Omidbaigi et al. 2008; Tian et al. 2009; Ebadollahi et al. 2010, 2011; Omidbaigi and Mahmoodi 2010; Skakovskii et al. 2010; Gong et al. 2012a, b; Li et al. 2013);
- Essential oil distillation–extraction (Wang 2010);
- Extraction with organic solvents: hexane, hexane–EtOAc mixtures, EtOAc, EtOAc–MeOH mixtures, MeOH, dichloromethane (Kim et al. 2001b; Shin et al. 2001; Estrada-Reyes et al. 2004);
- Extraction with diethyl ether and boiling methanol followed by cold storage (−20 °C) and steam distillation (Weyerstahl et al. 1992);
- Headspace (Mazza and Kiehn 1992; Wilson et al. 1992; Zielinska et al. 2011);
- Glass microneedles used for the determination of secretory trichomes constituents (Tirillini et al. 1997).

### Determination of non-volatile compounds

Plant material, such as aerial parts, roots and cell, tissue and organ cultures were extracted with various organic solvents of different polarities applied either independently or sequentially: n-hexane, petrol, petroleum ether, dichloromethane, chloroform, ethyl acetate, n-butanol, acetone, ethanol, methanol (two latter solvents also mixed with water) or water alone. Various extraction techniques were applied: maceration at ambient or elevated temperature, reflux extraction, infusions and decoctions in hot water. The extracts were usually dried under reduced pressure or by lyophilization (Itokawa et al. 1981; Ganeva et al. 1994; Lee et al. 1995, 2002, 2007, 2008; Kim et al. 1999; Molina-Hernandez et al. 2000; Suvitayavat et al. 2004; Vera-Montenegro et al. 2008; Xu et al. 2008; Hernandez-Abreu et al. 2009, 2011, 2013; Gonzalez-Trujano et al. 2012).

A few additional methods were used in a comparative study of the extraction of several herbs, including *A. foeniculum* (Dapkevicius et al. 1998):

- Deodorized acetone extract obtained by re-extracting all the dried solid retentate remaining after hydrodistillation with acetone under continuous shaking;
- Deodorized water extracts concentrated from the liquid retentate remaining after hydrodistillation;
- Supercritical CO_2_ extracts;
- Acetone extracts;
- Methanol–water extracts obtained by re-extracting the plant material remaining after the acetone extraction.

The headspace volatile organic compounds and the essential oil were usually analyzed using the gas chromatography mass spectrometry technique (GC–MS). For quantitative analysis, gas chromatography was used with a flame ionization detector (GC–FID) (Charles et al. 1991; Mazza and Kiehn 1992; Weyerstahl et al. 1992; Wilson et al. 1992; Svoboda et al. 1995; Dung et al. 1996; Tirillini et al. 1997; Kim et al. 2001; Estrada-Reyes et al. 2004; Mallavarapu et al. 2004; Bruni et al. 2007; Mo et al. 2009; Tian et al. 2009; Ebadollahi et al. 2010; Omidbaigi and Mahmoodi 2010; Wang 2010; Mo and Ma 2011; Gong et al. 2012a; Li et al. 2013; Lim et al. 2013). Non-volatile analysis was usually made with reversed phase HPLC using C18 silica columns eluted with acetonitrile/water or methanol/water mixtures, with photometric detection. Individual isolated compounds were identified by routine spectroscopic methods such as ^1^H and ^13^C NMR, UV/VIS, infrared, and mass spectrometry (RA—Kim et al. 1999; acacetin, tilianin, agastachoside, linarin—Zakharova et al. 1980; thymol, myrcene, limonene, β-caryophyllene, pulegone, menthone, isomenthone, camphor, linalyl acetate, linalool, β-phellandrene—Skakovskii et al. 2010; acacetin, tilianin, isoagastachoside, agastachin—Itokawa et al. 1981; Hernandez-Abreu et al. 2009; agastinol, agastenol—Lee et al. 2002, agastaquinone—Lee et al. 1995).

The compounds were mostly purified using column chromatography on silica gel eluted with various solvents, sometimes preceded by liquid–liquid extraction:

- Chloroform–methanol mixture, methanol–water mixture, chloroform–methanol mixture, methanol (RA—Kim et al. 1999; tilianin–Hong et al. 2001; agastinol, agastenol—Lee et al. 2002);
- Petroleum ether-acetone mixture (estragole, methyleugenol—Li et al. 2013);
- Ethyl acetate/n-hexane gradient n-hexane/chloroform/methanol (for the elution of fractions) (agastaquinone—Lee et al. 1995);
- Hexane–ethyl acetate (*l*-pulegone—Maruyama et al. 2002, Estrada-Reyes et al. 2004; estragole, triterpenoids, flavonoids—Estrada-Reyes et al. 2004).

Further purification steps included column chromatography on different stationary phases, like C18 silica gel or Sephadex LH-20, sometimes finalized by preparative reversed-phase HPLC (on C18 columns).

In bioactivity or environmental-screening studies, simple colorimetric assays were used for the determination of the total content of certain metabolite classes, like polyphenols (with a Folin-Ciocalteu reagent), hydroxycinnamic acids (the European Pharmacopoeia method) and flavonoids (with aluminum chloride) (Suchorska-Tropiło and Pióro-Jabrucka 2004; Ibarra-Alvarado et al. 2010).

### Essential oil composition

Typically for *Nepetoideae*, the volatiles produced by *Agastache* plants are synthesized and stored in glandular trichomes localized in crypts on the leaf surface. These specialized morphological structures appear during leaf development and are fully differentiated in mature organs. Apart from the glandular trichomes, the leaf lamina of different *Agastache* species is more or less covered with non-secreting hair. In some species, like *A. foeniculum*, the hair cover is particularly dense (Svoboda et al. 1995). Depending on the species and plant organ, the oil yield can reach above 2 % (v/w) (the specific values are given in Table 2) (Charles et al. 1991; Svoboda et al. 1995; Omidbaigi and Sefidkon 2004; Omidbaigi et al. 2008; Omidbaigi and Mahmoodi 2010). In most of the studies the essential oil was obtained by the hydro-distillation of aerial parts, either all together (herb) or separated into leaves and inflorescences (Charles et al. 1991, Svoboda et al. 1995; Dung et al. 1996, Omidbaigi and Sefidkon 2003, 2004; Mallavarapu et al. 2004; Omidbaigi et al. 2008; Ebadollahi et al. 2010; Omidbaigi and Mahmoodi 2010; Gong et al. 2012a, 2012b; Li et al. 2013).

**Table 2:** Yield of essential oil (from dried herbal material) obtained from different *Agastache* species

Species	Oil yield (% v/w) d.w.	References
*A. foeniculum*	0.07–3.00	Charles et al. (1991)
	0.02–0.74	Svoboda et al. (1995)
	1.87	Omidbaigi and Sefidkon (2003)
	1.5–1.8	Mallavarapu et al. (2004)
	2.0	Omidbaigi and Sefidkon (2004)
	0.5–0.8	Suchorska-Tropilo and Pióro-Jabrucka (2004)
	2.1–2.88	Omidbaigi et al. (2008)
	2.3	Omidbaigi and Mahmoodi (20100
*A. mexicana*	1.45	Svoboda et al. (1995)
	0.4–0.6	Suchorska-Tropilo and Pióro-Jabrucka (2004)
*A. rugosa*	1.53–2.73	Charles et al. (1991)
	0.92–2.28	Svoboda et al. (1995)
	0.5–0.8	Dung et al. (1996)
	0.19	Maruyama et al. (2002)
	0.3–1.0	Suchorska-Tropilo and Pióro-Jabrucka (2004)
	0.37	Wang (2010)
	0.29–0.57	Gong et al. (2012a)
	0.32	Li et al. (2013)
*A. scrophulariifolia*	0.99	Svoboda et al. (1995)
*A. urticifolia*	0.89	Svoboda et al. (1995)

The content of essential oil in the herb of different *Agastache* species depends upon the time of harvest as well as on the environmental conditions or methods of cultivation (Omidbaigi and Mahmoodi 2010; Omidbaigi et al. 2008; Omidbaigi and Sefidkon 2004; Suchorska-Tropiło and Pióro-Jabrucka 2004; Svoboda et al. 1995). For example, the highest oil yield from *A. mexicana*, and *A. rugosa* was at the beginning, whereas from *A. foeniculum* in the middle of blooming period (Suchorska-Tropiło and Pióro-Jabrucka 2004). *A. rugosa* and *A. scrophulariifolia* grow well and give a higher yield of essential oil in cooler summer temperatures. Conversely, *A. foeniculum* and *A. urticifolia* prefer warmer conditions, otherwise they do not bloom and produce only negligible amounts of oil (Svoboda et al. 1995; Rudik 2013).

Moreover, plants from five *Agastache* species—*A. foeniculum, A. mexicana, A. rugosa, A. scrophulariifolia,* and *A. urticifolia* (Svoboda et al. 1995)—produced significantly more essential oil during the flowering phase than during vegetative growth. The yield decreased again before senescence time.

The sowing time is also an important factor influencing both the quantity and quality of essential oil from *A. foeniculum* (Omidbaigi and Sefidkon 2004). Early sowing (March) was advantageous over later months (May, July) resulting in higher yields (2 %) and estragole content (92 %). The same research group determined the positive effect of nitrogen fertilization, which could improve the essential oil yield by up to 2.88 % (at 100 kg N/ha). The moderate irrigation of fields in the vicinity of Tehran (Iran) was also beneficial (the highest yield was ca. 2.3 %), but this treatment does not need to be equally useful for areas with less arid conditions during summer (Omidbaigi et al. 2008; Omidbaigi and Mahmoodi 2010).

**Fig. 1:**
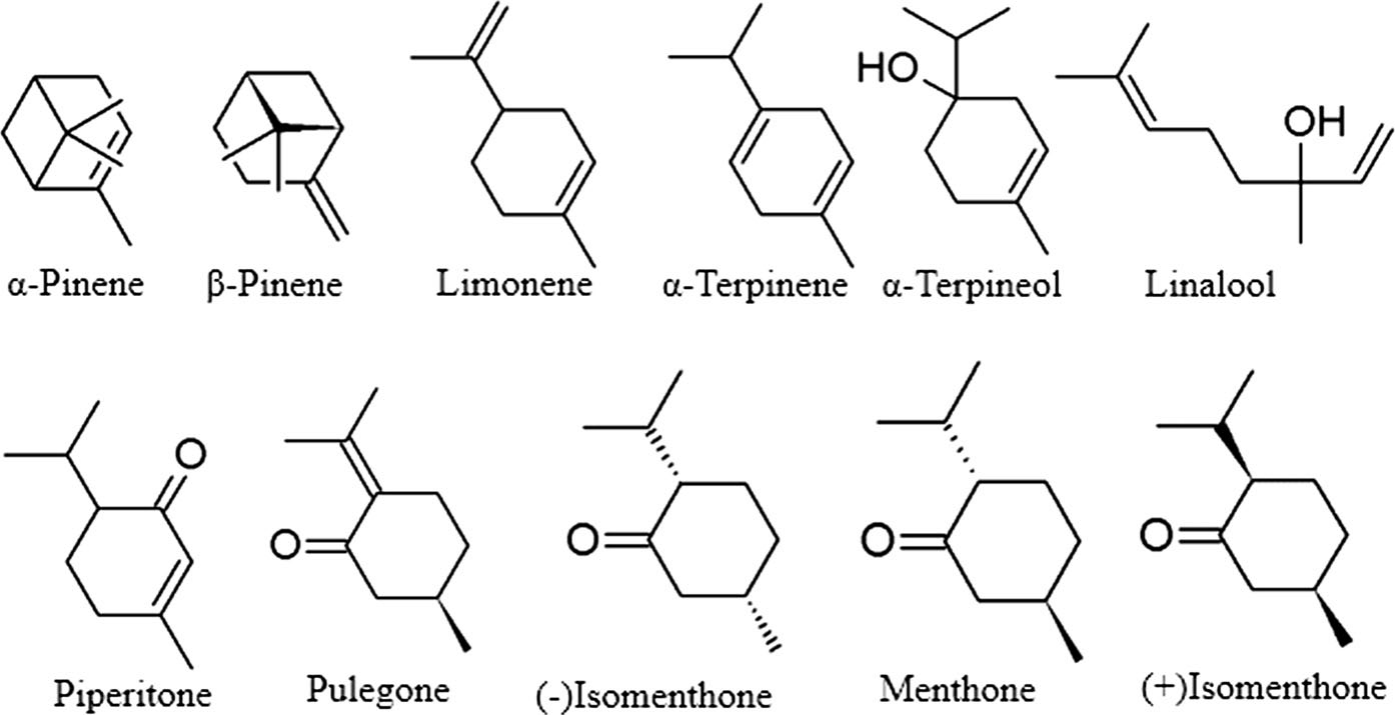
Structures of typical monoterpenoids from *Agastache*

**Fig. 2:**
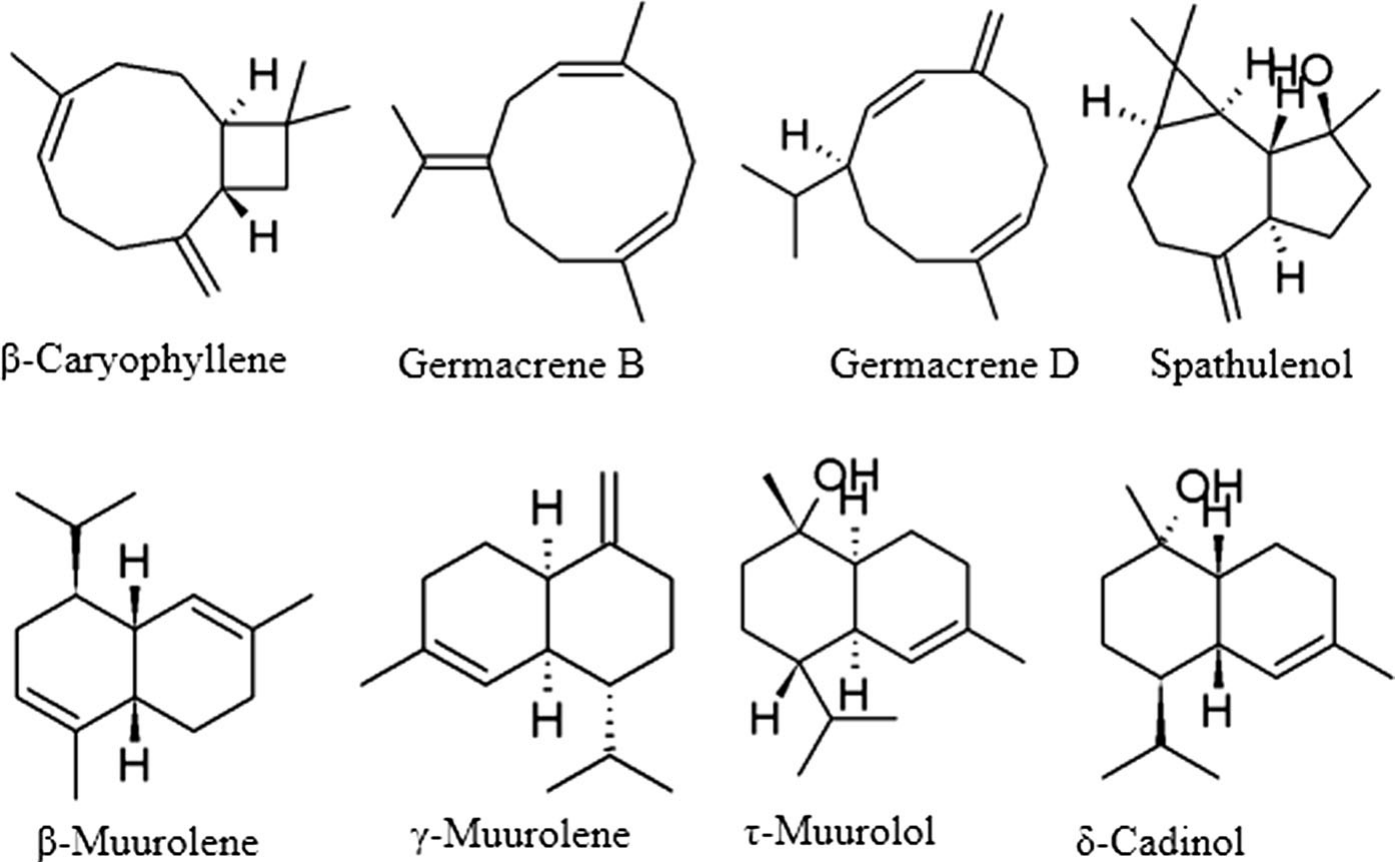
Sesquiterpenoids from *Agastache*

**Fig. 3:**
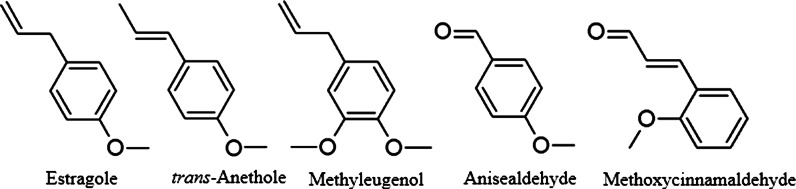
Structures of volatile phenolic compounds from *Agastache*

Although the composition of volatiles from the *Agastache* species is relatively variable, some of the constituents are usually predominating or else comprise a substantial fraction (Figs. 1, 2, 3). However, there are some discrepancies in the results of particular reports on several species as regards the stability of composition and the influence of environmental or hereditary factors.

Two main classes of volatile compounds are—phenylpropanoids and terpenoids. The latter comprise more abundant monoterpenes (hydrocarbons and oxidized) and sesquiterpenes, usually present only in small amounts.

Estragole confers the most typically-described anise-like aroma to the plants and essential oils of *A. rugosa*, *A. foeniculum* and *A. mexicana* (Fuentes-Granados et al. 1998). Estragole (syn.: methyl chavicol, p-allylanisol, 4-metoxyallylbenzene) is usually the most abundant constituent (18.6 % to over 98 %) in *A. rugosa* and *A.* *foeniculum* aerial parts (Fujita and Fujita 1973; Charles et al. 1991, 1992; Mazza and Kiehn 1992; Weyerstahl et al. 1992; Wilson et al. 1992; Svoboda et al. 1995; Dung et al. 1996; Tirillini et al. 1997; Omidbaigi and Sefidkon 2004; Skakovskii et al. 2010; Wang 2010; Zielinska et al. 2011; Lim et al. 2013). In essential oils from other *Agastache* species (*A. mexicana*, *A. mexicana subsp. xolocotziana* and *A.* *scrophulariifolia*) estragole was reported in various proportions (from absent to over 86 %) (Svoboda et al. 1995; Estrada-Reyes et al. 2004; Suchorska-Tropilo and Pióro-Jabrucka 2004). Other phenylpropene volatiles are also present in varying amounts in *Agastache*. There are populations and individuals that are distinct in their essential oil compositions from the most commonly described. For example, the closest biosynthetic relative of estragole—methyleugenol—was a main compound (over 85 %) in some specimens from Japan (Fujita and Fujita 1973), China (Li et al. 2013), and in Korean accessions (Chae et al. 2005). The latter authors suggest the existence of five chemotypes, based on the analysis of specimens of various geographical origins, cultivated in similar conditions: 1—the typical estragole-containing one (distributed throughout the species range), and four others (limited in their occurrence to one province)—with major constituents of headspace volatiles being: 2—menthone, 3—menthone and pulegone, 4—methyleugenol, and 5—methyleugenol and limonene. In *A. rugosa*, essential oil samples from Western China, Gong et al. (2012a, b) reported large amounts (48.8 and 19.2 %) of *p*-menthan-3-one ((−)isomenthone), estragole (20.8 and 29.5 %) and monoterpenes (8.8 %). Estragole was not detected in the essential oil of *A. mexicana* cultivars or *A. scrophulariifolia*, while the samples were rich in pulegone, at 75.3 and 45.2 % respectively (Svoboda et al. 1995; Estrada-Reyes et al. 2004). *A. foeniculum* can also contain up to 19.6 % (*E*)-anethole, as the second most abundant compound after estragole (59.5 %) in directly-sampled (with microneedle) secretory trichomes (Tirillini et al. 1992). However, anethole and other phenylallyls were usually detected in *Agastache* sp. only as minor compounds (Fuentes-Granados et al. 1998; Zielińska et al. 2011). In comparative analyses of 15 *A. foeniculum* populations, those of them with a low estragole content were high in sesquiterpenoid spathulenol (10.5–49.5 %) accompanied by various amounts of bornyl acetate (Charles et al. 1991). Moreover, these accessions also had a markedly lower essential oil content (0.07–0.36 %) than estragole-rich ones (0.80–2.45 %). The significance of such relationships has not yet been clarified, but it could relate to some environmental (rather than hereditary) factors. Interestingly, sesquiterpenes (δ-cadinol, β-caryophyllene and spathulenol) were also predominant in *A. nepetoides*, analyzed during the same study and likewise poor in essential oil (0.18 %). One of the volatile monoterpenoids that is produced in a higher proportion by different species of *Agastache* is pulegone. It was reported in large amounts in the essential oil of *A. rugosa* (13.4–50.8 % (Svoboda et al. 1995; Maruyama et al. 2002; Mo et al. 2009; Mo and Ma 2011), *A. foeniculum* (22.6 %), *A. scrophulariifolia* (45.2 %) (Svoboda et al. 1995) and *A. mexicana subsp. xolocotziana* (80 %), but not in *A. mexicana subsp. mexicana* (Estrada-Reyes et al. 2004).

Major volatiles are usually accompanied by less abundant monoterpenes (e.g., *d*-limonene, *α*-pinene, *β*-pinene, γ-terpinene, *α*-terpineol, linalool, thymol, menthofuran) and sesquiterpenes (e.g., *β*-caryophyllene, carvacrol, germacrene B, germacrene D) (Polak and Hixon 1945; Fujita and Fujita 1973; Charles et al. 1991; Mazza and Kiehn 1992; Weyerstahl et al. 1992; Wilson et al. 1992; Tirillini et al. 1997; Maruyama et al. 2002; Omidbaigi and Sefidkon 2003; Mallavarapu et al. 2004; Zielinska et al. 2011; Li et al. 2013). Recently, several sesquiterpenoids (elixene, γ-muurolene, viridiflorol, τ-muurolol), previously unknown from this genus, have been reported in the essential oil of *A. rugosa* aerial parts obtained by hydrodistillation (Li et al. 2013). In a volatile fraction obtained by simultaneous distillation–extraction, β-muurolene was present in a considerable amount, in 1.42 % of the total volatile fraction (Wang 2010). The analysis of essential oils from the leaves and flowers of *A. foeniculum*, *A. rugosa* and putative hybrids between *A. rugosa* and *A. foeniculum* was reported by Charles et al. (1991) who examined 19 different accessions (11 of *A. foeniculum*, four of *A. rugosa* and four of *A. rugosa* x *A. foeniculum* putative hybrids). In seven samples from *A. foeniculum*, neither menthone nor isomenthone were detected. The other four contained a low percentage of isomenthone (0.14, 0.17, 0.88 and 1.12 %). By contrast, isomenthone (0.31 % to 3.33 %) was present in *A. rugosa* and putative hybrids between *A. rugosa* and *A. foeniculum*. These hybrids were identified by the leaf and inflorescence morphology (Charles et al. 1991). However, in another study on four populations of *A. foeniculum*, the content of isomenthone was more diverse, ranging from 0 to 37.1 % (Svoboda et al. 1995).

Infection by the cucumber mosaic virus (CMV) caused alteration in the composition of *A. foeniculum* essential oil (Bruni et al. 2007). The most significant differences were observed in the content of estragole, isomenthone, pulegone and limonene. The concentrations of limonene and isomenthone increased, respectively, from 2.8 to 12.0 %, and from 27 to 43.9 %, whereas estragole and pulegone decreased from 16.2 to 3.2 %, and from 31.2 to 18.7 %. A significant quantitative decline of the oil yield (3.5 ml/kg—healthy plants, 0.4 ml/kg—infected plants) was also observed (Bruni et al. 2007).

### Phenolic compounds

**Fig. 4:**
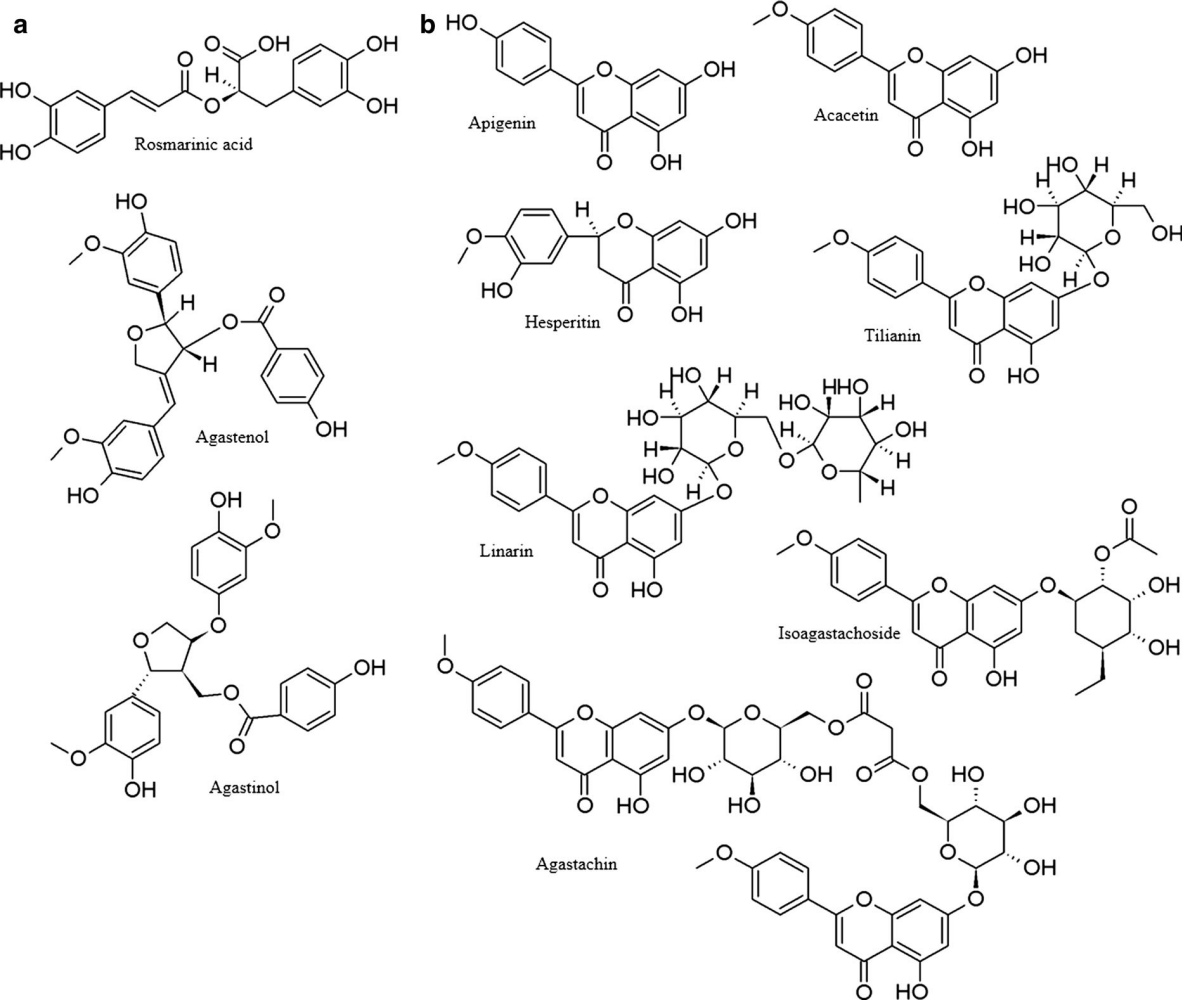
Non-volatile phenolic compounds from various *Agastache* species. **a** Phenylpropanoids. **b** Flavonoids

Caffeic acid derivatives—especially rosmarinic acid and several glycosylated flavonoids are the most abundant non-volatile phenolic metabolites in different *Agastache* species (Fig. 4). The content of both flavonoids and phenolic acids depends on the plant organ and the ontogenetic phase, and also on external factors such as biotic and abiotic stress and environmental conditions.

#### Flavonoids

One of the typical flavones reported in *Agastache* is an acacetin (5,7-dihydroxy-4′-methoxyflavone) glycoside—tilianin (acacetin-7-O-β-D-glucopyranoside) present in the aerial parts and roots of *A. rugosa* and *A. mexicana* (Itokawa et al. 1981; Zou and Cong 1991; Tuan et al. 2012; Hernandez-Abreu et al. 2013). This compound appears to be more representative for *A. mexicana* than for *A. rugosa*. The content of tilianin in *A. mexicana* aerial parts measured in various methanol extracts was over 8 mg/g of dried herb (Hernandez-Abreu et al. 2011). *A. rugosa* contains much lower amounts of tilianin, reaching 6.33 μg/g d.w. in flowers and even less in other organs (2.18 μg/g in leaves, 0.49 μg/g in stems, and 0.14 μg/g in roots) (Tuan et al. 2012). However, Hong et al. (2001) were able to isolate almost 50 g of pure tilianin from 30 kg of herb (yield of about 1.65 mg/g), which is still a significantly lower amount compared to the yield of *A. mexicana*. Other flavonoids detected in these two species belonged mainly to various subclasses of aglycones, such as hesperetin, apigenin, salvigenin, kaempferol and quercetin, as well as (+)-catechin in *A. mexicana* (Estrada-Reyes et al. 2004; Suchorska-Tropiło and Pióro-Jabrucka 2004; Ibarra-Alvarado et al. 2010), while apigenin, acacetin and 4′,5-dihydroxy-3,3′,7-trimethoxyflavone were in *A. rugosa* (Zakharova et al. 1980; Ishitsuka et al. 1982; Suchorska-Tropiło and Pióro-Jabrucka 2004).

Glycosides other than tilianin were also obtained from the aerial parts of *A. rugosa*: linarin (7-O-rutinoside of acacetin) and agastachoside (6″-*O*-acetyl-7-β-d-glucopyranosyloxy-5-hydroxy-4′-methoxyflavone) (Zakharova et al. 1980). Itokawa et al. (1981) were able to isolate not only two previously described flavonoids—acacetin (285 mg from1 kg of herb) and tilianin (46 mg), but also two new compounds—210 mg of isoagastachoside (2″-*O*-acetyl-7-β-d-glucopyranosyloxy-5-hydroxy-4′-methoxyflavone) and 19 mg of a quite unique malonyl diglucosylflavonoid, named ‘agastachin’ (di-(6″-acacetin-7-glucosyl) malonate). *A. pallida* (Lindl.) Cory var. *pallida* (identified by the authors as *A. barberi* (B.L. Rob.) Epling from *Brittonastrum* section, was analyzed for surface flavonoids in a comparative study of *Nepeta sp*. and related genera. In this plant, four flavones were detected—luteolin, acacetin, cirsimaritin (5,4′-dihydroxy-6,7-dimethoxyflavone) and isothymusin (5,8,4′-trihydroxy-6,7-dimethoxyflavone) (Jamzad et al. 2003). No quantitative data were provided, however.

Different contents of flavonoids were observed in the aerial parts of *A. rugosa*, *A. mexicana*, and *A. foeniculum,* at three different times of harvest during blooming (Suchorska-Tropiło and Pióro-Jabrucka 2004). A high content of flavonoids (apigenin, quercetin) was observed at the beginning of the flowering period or else at full bloom, while their concentration declined as senescence set in. The largest amount of apigenin (1.62 mg/g) was reported for *A. foeniculum* and the smallest (0.17 mg/g) for *A. rugosa* at the beginning of blooming. At full bloom, there was also a significant amount of quercetin (1.97 mg/g) in *A. foeniculum*. The content of total hydroxycinnamic acids was not dependent on the term of harvest.

#### Phenolic acids and lignans

While low concentrations of flavonoids like acacetin and tilianin were reported in *A. rugosa*, RA content observed during the same experiment was markedly higher (Tuan et al. 2012). The highest amount of RA was detected in flowers, where its content was 48.43 μg/g d.w., as well as in roots (30.97 μg/g) and leaves (22.14 μg/g). The lowest content of RA was reported in stems (9.14 μg/g). Janicsak et al. (1999) compared rosmarinic and caffeic acid contents in 96 *Lamiaceae* taxa, including three *Agastache* species. The RA level was over four times higher than that of caffeic acid in all three species, and was as follows: *A. mexicana*—0.64 versus 0.15 mg/g, *A. urticifolia*—0.30 versus 0.05 mg/g, and in *A. foeniculum*—0.27 versus 0.06 mg/g of rosmarinic and caffeic acids, respectively.

From the entire plant of *A. rugosa*, two lignans—agastinol and agastenol—were isolated and characterized (Lee et al. 2002). Both compounds are built of three aromatic moieties (two phenylpropanoid and one benzoyl) substituted by three hydroxy- and two methoxy- groups in the same positions. Agastenol is (7′*R*,8′*S*)-4-hydroxybenzoic acid 4-(hydroxy-3-methoxybenzylidene)-2-(4-hydroxy-3-methoxyphenyl)tetrahydrofuran-3-yl-methyl ester, whereas agastinol is a derivative with a saturated bond between carbons 7 and 8—(8*S*,7′*R*,8′*S*)-4-hydroxybenzoic acid 4-(4-hydroxy-3-methoxybenzyl)-2-(4-hydroxy-3-methoxyphenyl)tetrahydrofuran-3-yl-methyl ester.

### Non-volatile terpenoids and sterols

**Fig. 5:**
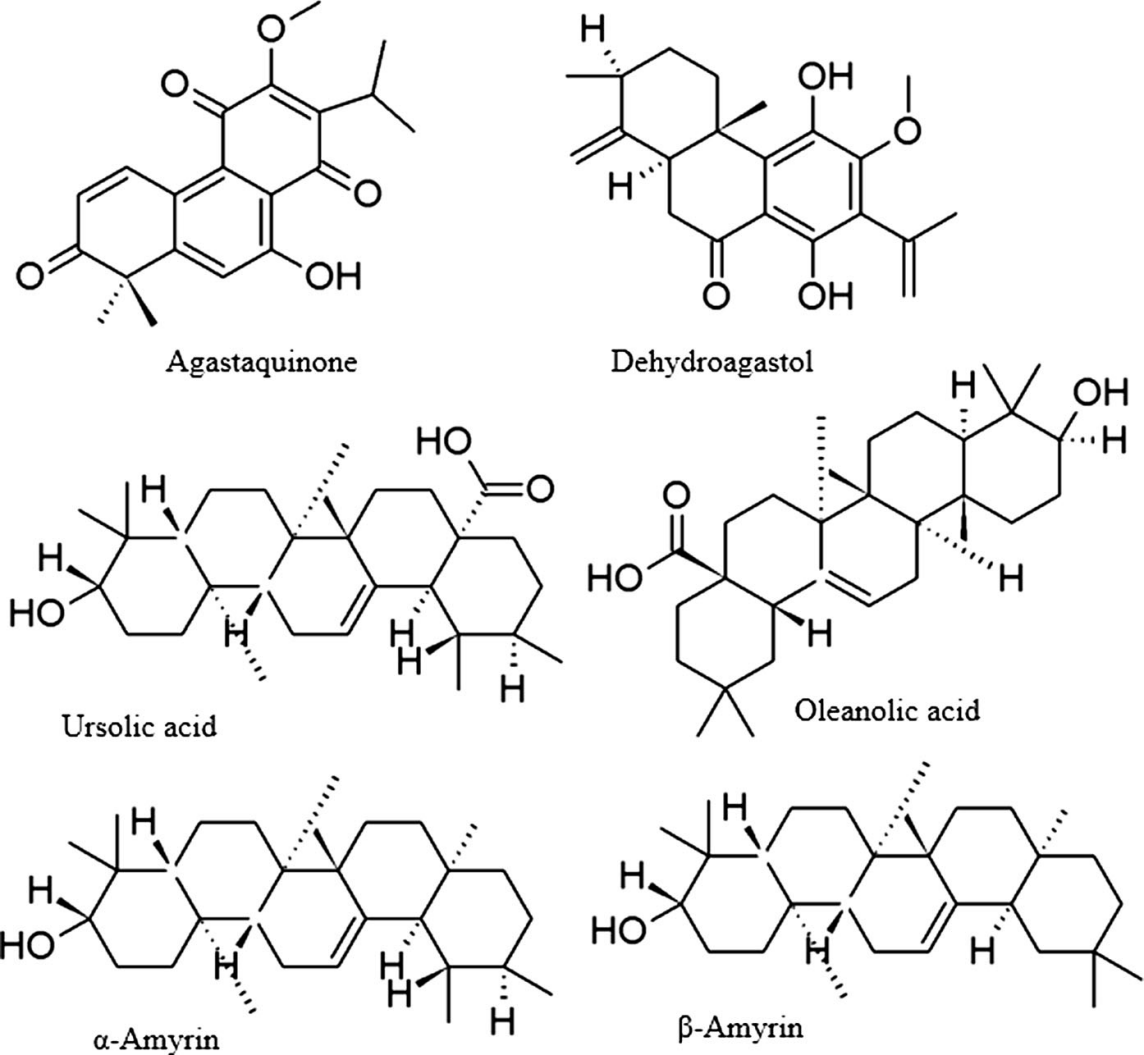
Structures of diterpenoids and pentacyclic triterpenes from *Agastache*

From *A. rugosa* roots, several new diterpenoids (examples in Fig. 5) were isolated and identified, such as a red-colored nor-abietanoid agastaquinone (Lee et al. 1995), and other oxidized abietanoids: agastol, dehydroagastol, isoagastol (Han 1987; Zou and Cong 1991), agastanone, and methylagastanol (Han 1987; Lee et al. 1994).

*A. rugosa* roots also contain several pentacyclic carboxylated and hydroxylated triterpenoids (Fig. 5) of oleanane-type (maslinic acid, oleanolic acid, 3-*O*-acetyl oleanolic aldehyde), ursane-type (corosolic acid) and the sterols β-sitosterol and daucosterol (Han 1987; Zou and Cong 1991; Estrada-Reyes et al. 2004). An ursane-type structure was also represented by ursolic acid isolated from aerial parts of *A. mexicana* with a 0.33 % yield (Verano et al. 2013). Both oleanane (β-amyrin) and ursane (α-amyrin) triterpenoids were isolated from *A. foeniculum* aerial parts, together with numerous sterols and stanols (campesterol, campestanol, sitosterol, stigmasterol, stigmastanol: Fig. 5) (Ganeva et al. 1994; Zou and Cong 1991). Given the ubiquitous presence of both pentacyclic triterpenoids and sterols in plants, they can also be expected in other *Agastache* species, but there are no qualitative or quantitative data available so far.

The seed oil from *A. rugosa* studied for fatty acid composition consisted of 91 % unsaturated fatty acids, of which 52 % were C18:3 (linolenic acid), 27.5 % were C18:2 (linoleic acid) and 11.5 % were monounsaturated C18:1 (oleic acid) acids (Zakharov et al. 1988).

As in every green plant, several carotenoids are also present in *Agastache*. Their quantitative analysis was performed in *A. foeniculum* and *A. rugosa* by means of RP-HPLC using a C30 column. β-Carotene was the most abundant followed by xanthophylls—lutein in amounts comparable to β-carotene and three less abundant—zeaxanthin, violaxanthin and antheraxanthin. *A. rugosa* contained larger amounts of each detected carotenoid—499.2 versus 260.9 μg/g of β-carotene and 277.1 versus 189.7 μg/g of lutein (Chae et al. 2013).

## Bioactivity of individual species as medicinal plants

Of the three most important medicinal species, *A. rugosa* is the main object in most of the published bioactivity data (Table 3). *A. rugosa* is the only species native to East Asia, and it is an important herbal drug in Chinese, Korean and Japanese traditional medicine. As such, it has been frequently studied for various pharmacological activities in both in vitro and animal models.

**Table 3:** Overview of biological activities of *Agastache* species

Species	Source plant part	Extract type/compound/fraction	Activity	Reference
*Agastache rugosa*	Roots	Agastaquinone (diterpenoid quinone), its oxime derivative	Nonspecific cytotoxicity against human cancer cell lines	Lee et al. (1995)
	Roots	Rosmarinic acid, rosmarinic and caffeic acids methyl esters	In vitro anticomplementary	Oh et al. (1996)
	Leaves	4′,5-Dihydroxy-3,3′,7-trimethoxyflavone	Antiviral against poliovirus	Sandoval and Carrasco (1997)
	Not reported	Essential oil	Antibacterial against skin bacteria	Depo et al. (1998)
	Roots	Rosmarinic acid	Antiviral against human immunodeficiency virus (anti HIV-1 integrase)	Kim et al. (1999)
	Aerial parts	Estragole	Antifungal	Błaszczyk et al. (2000)
	Aerial parts	Tilianin	Inhibition of TNF-α-induced expression of VCAM-1	Hong et al. (2001)
	Flowers	Essential oil, limonene, anise aldehyde	Anticancerogenic, antimutagenic, cytotoxic	Kim et al. (2001a)
	Aerial parts	Essential oil	Antibacterial, antifungal	Song et al. (2001)
	Whole plant extract	Agastinol, agastenol	Inhibition of caspase-3 induction in U937 leukemia cells	Lee et al. (2002)
	Whole plant	Whole plant methanolic extract	Insecticidal against *Lasioderma serricorne*	Kim et al. (2003a)
	Whole plant	Whole plant methanolic extract	Insecticidal against *Sitophilus oryzae* and *Callosobruchus chinensis*	Kim et al. (2003b)
	Aerial parts	Essential oil	Antifungal	Shin and Kang (2003)
	Aerial parts	Estragole, essential oil	Antifungal against *Trichophyton* sp.	Shin (2004)
	Aerial parts	Estragole	Antifungal against Candida	Shin and Pyun (2004)
	Aerial parts	Tilianin	Anti-atherogenic	Nam et al. (2005)
	Leaves	Leaf lyophilized water extract	Inhibition of iNOS expression and NO production in ROS 17/2.8 cells	Oh et al. (2005)
	Leaves	Tilianin	Antioxidant	Oh et al. (2006)
	Whole plant	Whole plant methanolic Extract	Source of mite control fumigants for *Dermanyssus gallinae*	Kim et al. (2007)
	Calyx	Essential oil	Antioxidant	Tian et al. (2009)
*A. mexicana*	Aerial parts	Tilianin, methanolic extracts	Antihypertensive (vasorelaxant), NO production stimulating	Hernandez-Abreu et al. (2009, 2011, 2013)
	Leaves	Aqueous extracts	Reduce fever, premenstrual symptoms	Cano Asseleih (1997)
	Leaves	Aqueous extracts	Antidepressant and anxiogenic	Molina-Hernandez et al. (2000)
	Whole plant	Aqueous extracts	Vasoactive, antioxidant	Ibarra-Alvarado et al. (2010)
	Inflorescences	Ursolic acid, acacetin	Spasmolytic, antinociceptive	Gonzalez-Trujano et al. (2012)
	Aerial parts	Ursolic acid	Antinociceptive	Verano et al. (2013)
*A. foeniculum*	Herb	Essential oil, water and carbon dioxide (supercritical) extracts	Antioxidant (β-Carotene bleaching)	Dapkevicius et al. (1998)
	Aerial parts, seeds, roots	Water extract	Antioxidant (FRAP)	Dragland et al. (2003)
	Aerial parts	Essential oil	Insecticidal against *Tribolium castaneum* larvae, *Rhyzopertha dominica*, *Oryzaephilus surinamensis*, and *Lasioderma serricorne*	Ebadollahi et al. (2010, 2013), Ebadollahi (2011)

In Traditional Chinese Medicine (TCM), there is an ambiguity as to the identity of *Agastache rugosa* as a herbal drug. The pinyin name—‘*huoxiang*’ (藿香)—meaning *Agastachis herba*, can refer either to *Agastache rugosa* or to *Pogostemon cablin* (Blanco) Benth. (as *Pogostemonis herba*, ‘*Guang huoxiang*’). The latter herb, also known as ‘patchouli’, originates from the south-east Asia (Malaysia and Indonesia), and is commonly used as a substitute for *Agastache rugosa* due to the similar functions defined by the TCM (Holmes 1997; Chen et al. 2008). Similarly, in traditional Japanese phytotherapy, both herbs are utilized under the name ‘Kakko’ (Itokawa et al. 1981). In fact, the chemical composition (and typical scent) of these plants is quite distinct (Holmes 1997; Hu et al. 2006; Wu et al. 2013). According to TCM theory, ‘huoxiang’ is classified among aromatic, damp-dissolving herbs. It is said to dispel damp, release ‘exterior’ and ‘summer-damp’, relieve nausea and vomiting, and cure fungal infections. The taste and thermal properties of ‘huoxiang’ are pungent (acrid) and slightly warm, and the channel affiliations enter spleen, stomach and lung. The essential oil of *A. rugosa* is used against fever, headache, stomach pain, morning sickness (combined with *Scutellaria*) and other gastrointestinal disorders (Dung et al. 1996; Lee et al. 2004; Hou and Jin 2005).

*A. mexicana* has been used in Mexican folk medicine for the treatment of hypertension, stress and anxiety (Cano Asseleih 1997; Ibarra-Alvarado et al. 2010; Gonzalez-Trujano et al. 2012). Other North American species are also used as botanical drugs and food. Their usage by Native American people was summarized in the previous review (Fuentes-Granados et al. 1998). Briefly, most of applications refer to both the aerial parts and roots of *A. foeniculum* as a remedy against cough, fever and heart conditions, as well as externally in treating burns. Also, *A. nepetoides* leaves are used by Cayuga externally for treating burns (from poison ivy) and itching. Other folk medicinal species include: *A. urticifolia*, used in Nevada against swelling, gastric disorders and colds, *A. pallidiflora* used by the Navajo for the treatment of coughs and fevers, and *A. scrophulariifolia* roots, used by the Meskwaki as a diuretic decoction.

### Antimicrobial activity

Antimicrobial activity is among the most frequently reported properties of essential oils from different medicinal plants. *Agastache* is no exception. However, the strength of antibacterial and antifungal activity was rather moderate in all the studied species.

Estragole isolated from *A. rugosa* was more efficient against human pathogenic fungi as a pure compound than as a crude essential oil. The antifungal activity of estragole was proven against *Aspergillus niger*, *A. flavus*, *Trichoderma viride*, *Candida albicans*, *C. utilis*, *C. tropicalis*, *Cryptococcus neoformans*, *Trichophyton mucoides*, *T. tonsurans*, and *Blastoschizomyces capitatus* (Shin 2004; Shin and Kang 2003). The minimum inhibitory concentration (MIC) obtained for purified estragole was 2.5 mg/ml for estragole and 5.0 mg/ml or more for the essential oil. These results are similar to those obtained from other *Lamiaceae* species, like *Mentha spicata* and *Ocimum sanctum* (MIC 2.5 and 5 % v/v, respectively), whereas the antimicrobial activity of *Cymbopogon sp.* (*Poaceae*) essential oils was reported to be approximately two orders of magnitude higher (MIC at 0.06 %) (Bansod and Rai 2008).

Despite these barely noteworthy values, interesting interactions were demonstrated between *A. rugosa* essential oil and pure estragole and two reference fungicidal antibiotics – the ketoconazole and amphotericin B. FIC indexes with ketoconazole were 0.19 for estragole and 0.16 for *A. rugosa* oil against *B. capitatus*, and 0.28–0.50 against *Candida sp*. (clearly indicating a synergistic interaction). In contrast, amphotericin B and estragole were antagonistic against *Candida sp*. (Shin and Pyun 2004).

*A. foeniculum* essential oil tested against *Aspergillus sp*. and *Fusarium solani* was markedly weaker than Iranian thyme (*Thymus kotschyanus*) and garden savory (*Satureja hortensis*) oils (Ownagh et al. 2010). *Agastache* oil had detectable fungistatic activity only at the highest concentrations—1 and 2 mg/ml, whereas thyme and savory oils were fungicidal even below 125 μg/ml. In brief, we can conclude that the existing data are not in favor of popular claims about the strong antimicrobial properties of essential oils from either *Agastache* or other, related herbs.

### Antiviral activity

4-Methoxycinnamaldehyde from *A. rugosa* essential oil was reported to express antiviral activity against the human respiratory syncytial virus (Wang et al. 2009). This compound effectively inhibited the cytopathic effect of the respiratory syncytial virus in a human larynx carcinoma cell line (IC_50_ of 0.055 μg/ml and a selectivity index of 898.2). It was able to inhibit viral entrance by interfering with viral attachment (IC_50_ of 0.06 μg/ml) and internalization (IC_50_ of 0.01 μg/ml). Moreover, 4-methoxycinnamaldehyde at 0.1 μg/ml had a high cytoprotective rate (90 %), while the same dose of Ribavirin (a prodrug used as a control substance) saw no activity of this kind. However, we must note that the 4-methoxycinnamaldehyde used in this study was purchased from a chemical company as a standard substance and was not obtained from *A. rugosa*. The authors mention this compound as an active principle of *A. rugosa* essential oil. This assumption was based on indirect citations from earlier publications, where phenylpropanoids such as estragole and cinnamaldehyde derivatives were studies as attractants of rootworm beetles (Metcalf and Lampman 1989). Hence, it is hardly possible to conclude that essential oil form *A. rugosa* can indeed become a practical source of this antiviral compound. In our own research using the GC–MS method on volatiles from this plant, 4-methoxycinnamaldehyde was never detected (Zielińska et al. 2011).

An efficient anti-picornavirus compound was isolated from the leaves of *A. rugosa*. It was isolated and investigated under the code name Ro-09-0179 by a Japanese subsidiary of Roche (Ishitsuka et al. 1982). Chemically, it is 4′,5-dihydroxy-3,3′,7-trimethoxyflavone, also known as ‘pachypodol’, a tri-O-methyl ether of quercetin. This highly lipophilic flavonol selectively inhibited several human pathogenic RNA viruses, such as rhinovirus, coxsackievirus and poliovirus, acting on viral plus-strand RNA replication (at average MIC_90_ of 0.3 μg/ml) (Ishitsuka et al. 1982). It specifically targets the Golgi complex and inhibits the processes associated with retrograde transport (Sandoval and Carrasco 1997).

Whether or not this promising compound has real therapeutic value and will be introduced as a drug or lead structure cannot be predicted at the moment.

HIV is another RNA human virus targeted by *A. rugosa*. Methanol extract, and from that isolated RA, inhibited HIV integrase enzymatic activity in vitro (IC_50_ of RA was 10 µg/ml) (Kim et al. 1999). However, it is rather difficult to expect the actual clinical relevance of RA as an anti-HIV compound. Its common occurrence in various plants and broad spectrum of activities renders it more of a model structure for further experiments on antiviral mechanisms than as an applicable preventive phytopharmaceutical.

### Anti-mutagenic and cytotoxic properties

Essential oil from the flowers of *A. rugosa* was reported to express anti-mutagenic activity in a dose-dependent manner when tested in Chinese hamster ovary AS52 cells. The AS52 cell line is a specially designed, genetically engineered, hypermutating in vitro system for testing mutations related to oxidative stress. The crude oil was more active (14, 68 and 75 % inhibition of mutagenicity at concentrations 0.2, 0.6 and 1.0 g/l, respectively) than any of the three individual components (estragole −11, 16 and 38 %; limonene 21, 30 and 43 %; anisaldehyde 8, 49 and 63 %). Interestingly, the less abundant constituent of the oil—anisaldehyde was more potent than the two major compounds, estragole and limonene (Kim et al. 2001a). In the same study, the oil was relatively less cytotoxic to human liver cell cultures, and was inhibiting just below 12 % of the untreated cell growth rate. On the other hand, a prominent inhibition of cancer cell line growth was observed, as well as the rapid augmentation of T and B cells’ proliferation induced by the crude oil, which was not obtained by any of the three individual components. It is not possible to conclude from these results whether a synergy exists between the three oil components or whether the observed effects are caused by other minor, more active compounds.

A cytotoxic effect towards several cancer lines (lung, ovarian, melanoma, CNS and colon cancer) was also demonstrated by agastaquinone—a non-volatile diterpenoid from *A. rugosa* roots (Lee et al. 1995). The efficacy expressed as ED_50_ differed for each cell line from 1.8 μg/ml (for neuronal cancer cells) to 12.8 μg/ml, whereas cis-platin ED_50_ was 0.5–1.0 μg/ml.

Interestingly, two lignans from the same plant—agastinol and agastenol—caused quite different effects in leukemia cells U937, in which protection against etoposide-induced apoptosis was observed (Lee et al. 2002). Agastenol activity was close to the standard anti-apoptotic agent—pyrrolidine dithiocarbamate (IC_50_ 11.4 μg/ml and 8.3 μg/ml, respectively)—whereas agastinol was considerably less effective (IC_50_ 15.2 μg/ml). Although anti-apoptotic activity had been tested on a malignant cell line, it should be explored further as being potentially useful in degenerative and aging-related disorders.

The above results indicate the very promising potential of *A. rugosa* constituents in chemoprevention, which was demonstrated by in vitro cell-based assays. However, more investigations (including in vivo ones) are needed to confirm such properties. In particular, some minor but more active compounds from both essential oil and non-volatile fractions should be considered.

### Antioxidant activity

Given the significance of *Agastache* *sp.* in traditional phytotherapy and economic botany in different regions, one might expect at least several papers describing a once very fashionable antioxidant activity. Quite surprisingly, unlike for many other related food and medicinal plants, little has been published about it.

Certainly, it can be assumed that the content of such potent antioxidants as RA and polyhydroxylated flavonoids should contribute to this type of activity. In this regard, a comparative mechanistic study using at least several complementary assays would be of particular interest. It should encompass different *Agastache* species and compounds isolated from them, followed by an attempt to elucidate the role of the individual constituents in the extracts’ properties.

In one of the few in vivo studies that have been published so far, a TCM preparation containing essential oil from *A. rugosa* was remarkably efficient in alleviating heat stress symptoms. In pigs kept at 40 °C, the herbal treatment reduced the malonyldialdehyde level and restored SOD and GPx activity to the control level. In a cell culture experiment involving separated components of the TCM preparation, the essential oil of *A. rugosa* herb was among the most efficient agents (at 100 and 200 μg/ml) in improving antioxidant status after heat stress, but the differences were not significant (Guo et al. 2011). Another in vitro cell-based system in which *A. rugosa* has been improving an antioxidant marker is the standard monocyte/macrophage line RAW264.7 (Oh et al. 2006). Here, a concentrated decoction (hot water extract, lyophilized and dissolved at 28 % concentration) efficiently induced the expression of HO-1—one of the isoforms of heme oxygenase (HO). This effect was concomitant with decreased hydrogen peroxide cytotoxicity upon *A. rugosa* extract treatment. The suggested mechanism involves the protein kinase G pathway, evidenced by the use of PKG inhibitors, which partially counteracted the *A. rugosa* extract’s cytoprotective activity. However, the contribution of direct H_2_O_2_ scavenging is also possible. Unfortunately, nothing is known about the particular extract constituents that may be responsible for this effect. According to Oh et al. (2006), it could be attributed to tilianin, but the authors did not check its content nor did they perform any standardization of the extract. Water extraction would most likely yield other phenolic compounds, such as caffeic acid derivatives, which could contribute to both direct antioxidant and heme oxygenase-activating mechanisms. The ability to scavenge reactive oxygen species—such as hydroxyl radicals—was also demonstrated in a screening study of 25 traditional Chinese herbs, among which a tincture (ethanol macerate) from *Agastachis herba* was moderately active with an EC_50_ of 4.4 µg/ml (Wang et al. 2006). Interestingly, *Agastache* extract was least reactive in a total polyphenol test (29.3 mg/g gallic acid equivalents) of those with an EC_50_ was below 10 µg/ml. Bearing in mind the inaccuracy of a ‘total content of…’ approach in bioactivity studies, it can be speculated that, in *A. rugosa*, there are other compounds at work, not just Folin-Ciocalteu reagent-reducing agents.

### Cardiovascular and anti-inflammatory effects

Tilianin is frequently regarded as a major bioactive compound, especially in two of the most popular species—*A. rugosa* and *A. mexicana*. Some studies have been performed using isolated tilianin, whereas in others it was a phytochemical marker in various extracts or fractions.

Both tilianin and extract from *A. rugosa* had a positive effect on the early stages of atherosclerosis pathophysiology (Hong et al. 2001). In experiments with mice on a high cholesterol diet, addition of 1 % of *A. rugosa* methanol extract reduced the total plasma cholesterol levels (from 1065 mg/dl down to 986 mg/dl). The cholesterol content of VLDL was lower, too, but the change was not statistically significant (Hong et al. 2001).

Treatment with tilianin (0.05 % in the diet) resulted in the reduction (to 56.6 %) of the lesion area in the aortic sinuses of hyperlipidemic mice. However, the results showed no significant differences between tilianin treatment and a high cholesterol diet for the tested groups in terms of total cholesterol and HDL levels. Also, the triglyceride content was lower after tilianin supplementation in the tested groups of animals, although the results were not statistically significant (Nam et al. 2005).

However, pretreatment with tilianin significantly suppressed (80 and 74 % at 10 and 100 μmol, respectively) the TNF-α-induced expression of vascular cell adhesion molecule-1 (VCAM-1) in the human umbilical vein endothelial cells (Hong et al. 2001). In murine peritoneal macrophages, tilianin inhibited NF-κB activation in a concentration-dependent manner (1–10 μmol), causing a decrease of pro-inflammatory cytokines’ (TNF-α and IL-1β) mRNA levels (Nam et al. 2005).

Tilianin was also obtained from *A. mexicana*, which is actually a more abundant source of it. The antihypertensive properties of *A. mexicana* used in folk herbal medicine in Mexico are attributed to tilianin content (Hernandez-Abreu et al. 2009). In a comparative study of various solvent extracts prepared from herbs dried at different temperatures, vasorelaxant ex vivo activity correlated with the amount of tilianin (Hernandez-Abreu et al. 2011). However, the tilianin potency in relaxing rat aortic contractions was significantly weaker than the reference drug carbachol (EC_50_ 104 µg/ml and 0.075 µg/ml, respectively).

The mechanisms of hypotensive and vasorelaxant properties involve the nitric oxide/cGMP pathway and potassium channel (Hernandez-Abreu et al. 2009). A hypotensive effect on both systolic and diastolic blood pressure was confirmed in vivo using a spontaneously hypertensive rat model in which the ED_50_ was 53.51 mg/kg body weight (compared to captopril 30 mg/kg) (Hernandez-Abreu et al. 2013). In the same study, no toxicity was found in the mouse model up to the dose of 1 g/kg, which warrants further investigation at the clinical level. However, the overall efficacy of using *A. mexicana* as an anti-hypertensive remedy can be disputed, as demonstrated by the study of aqueous decoctions from 10 herbs (Ibarra-Alvarado et al. 2010). Of the seven herbs that evoked relaxation, *A. mexicana* extract was the second weakest (24.9 % relaxation of pre-contracted rat aortic segments), whereas four of them (*Psittacanthus calyculatus*, *Dracocephalum moldavica*, *Prunus serotina*, *Chiranthodendron pentadactylon*) reached more than 60 % (Ibarra-Alvarado et al. 2010). This result actually agrees with those previously mentioned, where aqueous extracts were poor in tilianin. Thus, the most popular method for preparing herbal tea is rather unlikely to be efficient against cardiovascular conditions.

### Neurological activities

Three different solvent extracts of *A. mexicana* subsp. *xolocotziana* exhibited anti-nociceptive activity, demonstrated in an animal study using rats and mice (Gonzalez-Trujano et al. 2012; Gonzalez-Ramirez et al. 2012). In a set of complementary tests, hexane, ethyl acetate and methanol extracts from macerated inflorescences were compared. As a result, all the extracts diminished nociception in experimental animals. Differences in the extracts’ polarities were reflected by their composition and the kind of induced pain that was reduced in each test. Ethyl acetate extract (containing significant amounts of ursolic acid) was the most efficient in reducing behavioral responses to pain induced by formalin, especially in the inflammatory (second) phase, while hexane extract (non-polar compounds, like pulegone and oleanolic acid) decreased reactions to heat-induced pain. However, the inflammation symptoms caused by formalin were inhibited mainly by methanol extract, which is rich in flavonoids such as acacetin and tilianin. Acacetin, present in methanol extract (14.9 mg/g), showed the highest anti-nociceptive activity (ED_50_ of 2 mg/kg) while that for diclofenac was 12 mg/kg and that for ursolic acid was 3 mg/kg. Spasmolytic response was also observed for acacetin at a concentration of 3.5 µmol. Furthermore, the same papers report a lack of gastric toxicity with high doses (1 g/kg body weight) of extracts. The same extracts protected gastric mucosa against lesions caused by the oral administration of absolute ethanol. This study suggests a possible application of a complex preparation from this herb in alleviating various pain-causing disorders, and confirms the typical ethnomedicinal use of *A. mexicana* in such conditions.

Likewise, (*R*)-(+)-pulegone from the essential oil of *A. rugosa* (syn. *A. formosana*) also exhibited anti-nociceptive activity against heat- and chemically-induced pain in mice, as well as CNS-depressing and anticonvulsant effects (de Sousa et al. 2011). It is not known what the cellular mechanisms of such activity are, but some structural resemblance to menthol (a widely-used monoterpene targeting several mediators in pain receptors, including kappa-opioid) could guide further research into pulegone and *Agastache* essential oil (Kamatou et al. 2013).

The aqueous extract of *A. mexicana* leaves exhibited antidepressant-like activity (Molina-Hernandez et al. 2000). The results of three different tests (an Elevated plus-maze test, a forced swimming test and an open field test) revealed the anxiogenic-like effect of *A. mexicana* extract rather than anxiolytic-like activity. In the elevated plus-maze test, *A. mexicana* water extract reduced open-arms exploration; the results of the forced swimming test also showed no antidepressant-like effect of the extract (12.0 mg/kg body wt.), tested on rats’ behavior using pentylenetetrazole (15 mg/kg body wt.) and desipramine (32 mg/kg body wt.) as control substances. However, when it was administrated together with desipramine, it increased the antidepressant-like effect of the latter, similar to the effect of pentylenetetrazole and desipramine co-administration. The open field test revealed no sedative effect of water extract of *A.* *mexicana* at the doses used. Again, the study is in partial agreement with traditional folk indications for this plant, but it also argues against its uncritical use as an anxiolytic remedy.

### Biocidal activity

Somehow distinct from above described pharmacological and preventive properties are the pesticidal activities of various substances from the *Agastache* species. Some of the results presented below indicate the real applicative potential of as environmentally friendly, biodegradable crop- and foodstuff-protection products.

*A. rugosa* methanol extract obtained by maceration exhibits insecticidal activity against stored products pests, such as the beetle species *Lasioderma serricorne* (infesting tobacco products) (Kim et al. 2003a) as well as *Sitophilus oryzae* (rice weevil) and *Callosobruchus chinensis* (bean weevil) (Kim et al. 2003b).

The insecticidal properties against various stored product-infesting beetles were also evidenced by *A. foeniculum* essential oil. The pests efficiently targeted by estragole and 1,8-cineole-rich oil included: *L. serricorne* (saw-toothed grain beetle), *Oryzaephilus surinamensis* L. (a major pest of oilseeds, such as nuts, sunflowers and other foodstuffs), the red flour beetle (attacking mainly ground starch materials) *Tribolium castaneum* Herbst, and the lesser grain borer *Rhyzopertha dominica* F. (a pest of cereal grains) (Ebadollahi 2011; Ebadollahi et al. 2010, 2013). *A. rugosa* has also been suggested as one of the plants that may serve as an alternative source of mite control fumigants for *Dermanyssus gallinae* De Geer, an important blood-sucking poultry ectoparasite causing losses in flocks of egg-laying hens (Kim et al. 2007).

*A. rugosa* essential oil was also efficient in killing *Meloidogyne incognita*—a root-knot nematode. However, the tested plant represented a chemotype with over 50 % methyleugenol and just 8.55 % estragole (Li et al. 2013). Weak nematicidal activity was also exhibited by *A. rugosa* essential oil against the Pinewood nematode *Bursaphelenchus xylophilus* (Andres et al. 2012). *A. mexicana* methanol extract tested against parasitic flatworm (*Fasciola hepatica*) in a screening study on 19 herbal species from Mexico did not show any fasciolicide properties (Vera-Montenegro et al. 2008). With regard to crop protection, even the attractant properties of estragole and other phenylpropanoids from *Agastache*, could be utilized as semiochemical bait ingredients for controlling diabroticide rootworm beetles (Metcalf and Lampman 1989).

### Herbal drug formulae

‘Huoxiang’ (*Agastachis herba*) is one of the 50 fundamental herbs used in TCM. It can be utilized as a single drug or as an ingredient in the multicomponent formulations most commonly used by TCM and other East Asian traditional phytotherapy systems (Table 4).

**Table 4:** Examples of herbal formulae containing *Agastache rugosa*

Name of the formula	Plant material	Use/indications	Country	References
Ya-hom	Whole plant	Stomach discomfort	Thailand	Suvitayavat et al. (2004)
One of the 600 types of plant materials	Whole plant	Food uses	Taiwan	Chau and Wu (2006)
Sopoongsan	Not reported	Anti-inflammatory Anti-microbial Anti-allergy Anticancer activity In human skin	Korea	Lee et al. (2007)
Gan-lu-xiao-du-dan	Not reported	Chronic hepatitis	Taiwan (Republic of China)	Chen et al. (2008)
QWBZP	Leaves	Infantile diarrhea caused by rotavirus	China (P.R.C.)	Wu et al. (2010)

One noteworthy example of a multidrug formula containing *A. rugosa* is Gan-lu-xiao-du-dan, used in chronic hepatitis. This drug, consisting of 10 herbs, has been among the 10 most popular traditional Chinese medicines prescribed in Taiwan for hepatitis (Chen et al. 2008).

Another example of a Chinese patent medicine containing *A. rugosa* leaf water extract is Qiwei Baizhu Powder (QWBZP). Other ingredients in QWBZP include the roots of *Panax ginseng*, *Atractylodes macrocephala*, *Pueraria lobata*, *Saussurea costus*, *Glycyrrhiza uralensis* and a mushroom—*Poria cocos* (Wu et al. 2010). This herbal preparation is effective against HRV (human rotavirus) infection and has been studied in baby mice who suffered from diarrhea. In this study, QWBZP extract stimulated the gene transcription of several interleukins and IFN-γ in intestinal mucosa epithelial cells, as well as modulated CD8 T cell subset density. The effect of the preparation was comparable to the standard antiviral drug Ribavirin.

There is also a traditional Korean formula—called ‘Sopoongsan’—which contains *A. rugosa* among 12 ingredients. Sopoongsan is composed of water or ethanol extracts from various plant species (besides *A. rugosa*, these are *Nepeta japonica*, *Glycyrrhiza uralensis*, *Panax ginseng*, *Cnidium officinale*, *Peucedanum japonicum*, *Dendrobium nobile*, *Angelica koreana*, *Citrus unshiu*, *Magnolia officinalis*), one mushroom species (*Poria cocos*) and one insect species—silk moth (*Bombyx mori*). This oriental medicinal prescription has been reported to exhibit anti-inflammatory, anti-microbial, anti-allergy and anticancer activity on human skin (Lee et al. 2007).

In Thailand, one of the folk herbal drug formulae used for the treatment of stomach discomfort is Ya-hom. It contains the entire plant of *A. rugosa* among 16 other plant substances (Suvitayavat et al. 2004). The ability of this formula to inhibit gastric acid secretion was evidenced in rats treated with histamine or carbachol (a synthetic acetylcholine derivative). Ya-hom treatment decreased pepsin and protein secretory rates and inhibited histamine-induced gastric mucosal blood flow. The effect was observed in a time- and dose-dependent manner. Moreover, Ya-hom enhanced visible gastric mucus secretion. The above effects support the use of this preparation against stomach discomfort (Suvitayavat et al. 2004). The research on composite preparations is not as advanced as on individual herbs or phytochemicals, due to its complexity and difficulties in reliable data analysis. However, the results obtained to date suggest the important contribution of *A. rugosa* to therapeutic properties of such preparations widely used in Asian phytomedicine.

## Biosynthesis of active compounds

The molecular level of the biosynthesis of specialized metabolites of *Agastache* has so far been considered in only two published articles, both using *A. rugosa*. The earlier one (Maruyama et al. 2002) reported cloning and the functional expression of an enzyme responsible for the production of a monoterpene (limonene), while the more recent one (Tuan et al. 2012) described the expression of several key genes for early, committed steps of the biosynthesis of phenolic compounds.

The *d*-Limonene synthase gene from *A. rugosa* was cloned using a polymerase chain reaction (PCR) based on the highly conserved sequences among terpene synthases (TSs) (Maruyama et al. 2002). Most of the synthase genes that had previously been discovered in various species of the *Lamiaceae* family encoded for the *l*-limonene synthases, and only one—from *Schizonepeta tenuifolia*—for the *d*-enantiomer (Maruyama et al. 2002). *A. rugosa* is a proper model for the characterization of *d*-limonene synthase, since the plant produces *d*-limonene and its downstream biosynthetic derivatives *l*-pulegone and *l*-isomenthone. The complete *A. rugosa* limonene synthase gene (*Ar*-*lms*) is a sequence of 2,077 nucleotides, and contains a 1,839 bp translated region encoding 613 amino acids. The deduced amino acid sequence contains a putative plastid-targeting fragment at its N-terminus. The recombinant *A. rugosa* limonene synthase gene was functionally expressed using an in vitro cell free-transcription/translation system. The recombinant protein yielded *d*-limonene as a single product of the enzymatic reaction from GPP (geranyl pyrophosphate). *A. rugosa* limonene synthase showed a high homology to the synthases (*d*- and *l*-limonene synthases) of closely-related species from the same plant family, indicating only a few amino acids’ residues that are the responsible for the stereochemistry of the enzyme. The highest homology (87.3 %) was found with the *d*-limonene synthase from *Schizonepeta tenuifolia*, and the molecular weight (60 kDa) of both proteins was identical. The deduced amino acid sequence of the *d*-limonene synthase gene from *A. rugosa*, exhibits a relatively high homology to the *l*-limonene synthase of other *Lamiaceae* species, such as: *Mentha spicata* (70.8 %), *M. longifolia* (70.6 %), *Perilla frutescens* (62.8 %) and *P. citriodora* (62.7 %). The comparison between *d*- and *l*-limonene synthase amino acid sequences from several *Lamiaceae* species indicated 11 amino acid residues (four aromatic and seven acidic) as candidates for controlling the stereospecificity of the enzymatic reactions. The transcription of the genes involved in the early steps of phenylpropanoid biosynthesis was examined in *A. rugosa* using quantitative real-time PCR (Tuan et al. 2012). The expression patterns of these enzymes (phenylalanine ammonia-lyase (PAL), cinnamate 4-hydroxylase (C4H), 4-coumarate: CoA ligase (4CL), chalcone synthase (CHS) and chalcone isomerase (CHI)) were correlated to the RA and tilianin contents in different organs.

The cDNA of two genes encoding the enzymes (chalcone synthase and chalcone isomerase) involved in the biosynthesis of tilianin in *A. rugosa* were also isolated and characterized (Tuan et al. 2012). CHS and CHI have demonstrated a high homology with respect to the enzymes of other species. For instance, the *A. rugosa* CHS amino acid sequence has a 95 % identity and 98 % similarity with *Perilla frutescens* CHS (*Lamiaceae*), a 95 % identity and 98 % similarity with *Solenostemon scutellarioides* CHS (*Lamiaceae*), a 93 % identity and 96 % similarity with *Misopates orontium* CHS (*Plantaginaceae*), and a 92 % identity and 96 % similarity with *Mazus pumilus* CHS (*Phrymaceae*). The results of homology analysis indicated four *A. rugosa* amino acid residues defined for the active sites (Cys 164, Phe 215, His 303 and Asn 336) that are conserved in all the identified chalcone synthases. The *A. rugosa* chalcone isomerase was highly homologous to *Perilla frutescens* CHI (79 % identity and 88 % similarity), *Scutellaria baicalensis* CHI—*Lamiaceae* (78 and 89 % similarity), *Camelia sinensis* CHI—*Theaceae* (75 % identity and 88 % similarity), and *Dianthus caryophyllus* CHI—*Caryophyllaceae* (74 % identity and 87 % similarity). In the *A. rugosa* CHI, four residues were found (Thr 60, Tyr 118, Asn 125 and Ser 202) that had already been known as active sites of chalcone isomerases.

The comparative analysis of the gene transcription level of biosynthetic enzymes and the content of secondary metabolites (acacetin, tilianin and RA) showed the constitutive expression of the genes in all organs. The highest transcript levels of *ArPAL* and *Ar4CL* were found in leaves, whereas *ArC4H*, *ArCHS* and *ArCHI* reached their highest expression in flowers. In roots, all five genes were expressed at a relatively low level (Tuan et al. 2012). These results reveal a complex regulation mechanism of the biosynthesis of *A. rugosa* flavonoids (acacetin, tilianin) and RA. The highest expression (= transcript accumulation) of *A. rugosa* *PAL* and *4CL* in leaves as well as *C4H* in flowers corresponded to a relatively high content of RA in those plant organs. Yet, a significant amount of RA was present in the roots, where the transcript levels of *PAL*, *C4H* and *4CL* were low. Mechanisms for the allocation of specific metabolites and metabolite fluxes within the plant body require more studies, which should focus especially on more downstream biosynthetic enzymes and genes, as well as on possible transport between tissues and organs.

### *Agastache* in in vitro cultures and the production of secondary metabolites

Plant in vitro cultures have been applied mainly to *A. rugosa* and only rarely to other species (two reports on *A. foeniculum*) for such purposes as micropropagation (Mazur and Reshetnikov 2005; Kayani et al. 2013; Zielińska et al. 2011) and as a model system for studying the biosynthesis regulation of volatile (Menghini et al. 1992; Shin et al. 2001; Zielińska et al. 2011) and non-volatile specialized metabolites, as well as the efficient production of desired compounds such as RA (Mazur et al. 2008; Xu et al. 2008; Kim et al. 2013).

Different organ and tissue cultures were initiated, including callus from various explants, suspension cell cultures and organs (shoots and roots). Hairy roots were also induced by transformation with *Agrobacterium rhizogenes*.

For the in vitro shoot multiplication of *A. rugosa*, 6-bezylaminopurine was the favored cytokinin for inducing a morphogenetic response, and stem nodal segments were the optimal explants for forming multiple shoots (Mazur and Reshetnikov 2005; Zielińska et al. 2011). Furthermore, the total shoot number per explant was higher when the proliferation medium was supplemented with a natural auxin (indole-3-acetic acid) rather than a synthetic auxinic herbicide (picloram) (Zielińska et al. 2011).

A micropropagation protocol using shoot multiplication from nodal explants, followed by rooting and acclimation, was also established for *A. foeniculum* (syn. *A. anisata*) (Kayani et al. 2013). As with *A. rugosa*, the best performance (eight shoots per explant) was achieved on a BA- and IAA-supplemented MS medium. Great diversity was observed in the headspace volatile metabolites’ profiles in *A. rugosa* shoot cultures (Zielińska et al. 2011). The composition of the volatiles emitted from the shoots growing on media supplemented with exogenous plant growth regulators was significantly different from the intact plant phytochemical profile. Under in vitro conditions, monoterpenes such as limonene, pulegone, menthone and isomenthone predominated, whereas estragole was present only in trace amounts. The estragole content in in vitro-germinated seedlings and intact plants was significantly higher (21 and 95 %, respectively). On the other hand, the content of individual monoterpenes also varied between different hormonal treatments. For example, pulegone constituted 12.2–92.7 % of the total headspace volatiles analyzed by SPME–GC–MS, isomenthone from 0.1 to 26.4 %, and *α*-pinene (which was absent from conventionally grown plants) from 1.6 to 25.9 %. These results show that in vitro conditions can greatly influence the proportions between phenylallyls and monoterpenes, as well as between different monoterpenes (Zielinska et al. 2011). In contrast, the shoot cultures of *A. foeniculum* produced mainly estragole but only traces of another volatile phenylpropanoid—*trans*-anethole. The culture media’s supplementation with the precursor of a general phenylpropanoid pathway—shikimic acid—enhanced the concentration of methyl chavicol, and several pigments (i.e., chlorophyll a, chlorophyll b and carotenoids). No direct correlation between estragole content and pigment concentration was observed (Menghini et al. 1992).

In a suspension cell culture of *A. rugosa* initiated from a leaf-derived callus maintained in the dark, hydrocarbons and sesquiterpenes were mostly identified, whereas elicitation with methyl jasmonate or jasmonic acid (both at 100 µM) and illumination with white light resulted in more monoterpenes and a low amount of estragole (0.58 %) (Shin et al. 2001). The results of another study (Kim et al. 2001b) also show that non-differentiated cells differ in the volatile metabolite production profile. In the suspension cultures also obtained from leaf calluses, 3-hydroxy-2-butanone was the most abundant compound, followed by 2,4,5-trimethyl-3-oxazoline, 1,2,4-trimethylbenzene and 1,3-butanediol. Estragole and other typical compounds of the species were not detected. In plants regenerated via in vitro cultures, the content of secondary metabolites (such as total phenolics, flavonoids, acacetin and tannins) also deviated from conventionally grown plants (Mazur et al. 2008).

*A. rugosa* suspension cultures maintained on media containing 2,4-D (0.5 mg/l) and kinetin (0.1 mg/l) produced only low amounts of RA (Kim et al. 2001c). The highest content of RA (slightly exceeding 0.4 mg/g d.w.) was obtained on the eighteenth day during the stationary phase of the culture in the cells, but not in the culture medium. Better results for RA production in suspension cultures of *A. rugosa* were obtained by treatment with benzothiadiazole combined with a yeast elicitor. The experiments with elicitation enhanced the production of RA in the suspension cells by a factor of 11.4, but the maximum content of this compound reached only 5 mg/g of dry weight. The benzothiadiazole alone had no effect on RA production. However, in later experiments similar suspension cultures were able to accumulate much higher quantities of RA. The best cell growth (7.0 g/l) and RA production (10.7 mg/g d.w.) were observed on a MS medium supplemented with BAP (0.1 mg/l) and with 2,4-D (2 mg/l). A slightly higher content for the RA (11.5 mg/g d.w.) was achieved after culturing the suspension cells on B5 (Xu et al. 2008).

In the most recent report, the elicitation of cell cultures with methyl jasmonate (50 µM) resulted in a more than five-times higher accumulation of RA, up to 36.58 mg/g (Kim et al. 2013), which was similar to the value obtained—for example—in *Lavandula vera* (33.2 g/l d.w.) (Georgiev et al. 2004).

A metabolomic and transcript analysis of the key biosynthetic genes encoding the enzymes for early committed steps in phenylpropanoid metabolism, such as phenylalanine ammonia lyase (*ArPAL*), cinnamate-4-hydroxylase (*ArC4H*), and 4-coumarate: coenzyme-A (CoA) ligase (*Ar4CL*), was published recently by Kim et al. (2013). The experiment using *A.* rugosa cell cultures elicited with methyl jasmonate demonstrated that both phenolic acids (*p*-coumaric, *p*-hydroxybenzoic, ferulic, rosmarinic) and precursors for the phenylpropanoid biosynthetic pathway (such as aromatic amino acids and shikimate) were induced as a response to MeJA treatment. The increased RA accumulation in the elicited cells might be due to the activation of the phenylpropanoid genes *ArPAL*, *ArC4H* and *Ar4CL*, whose transcripts were increased by a factor of 9.2, 7.8 and 7.4, respectively (Kim et al. 2013). Hairy roots were induced by the infection of the leaves and stem sections of *A. rugosa* with the *Agrobacterium rhizogenes* B1000 strain. The maximum biomass growth of 14.1 g/l in a basal liquid MS medium was achieved after 2 weeks of culture. In the hairy root cultures, the RA content reached 116.3 mg/g dry weight after 14 days of culture (Lee et al. 2008). It was significantly higher than the best results obtained from cell cultures and the roots of *A. rugosa*. The efficiency of this system is comparable to other representatives from *Lamiaceae*, like *Hyssopus officinalis*, *Ocimum basilicum*, *Salvia miltiorrhiza* and *S. officinalis*, usually reaching between 5 and 15 % of dry weight (reviewed by Park et al. 2008).

### Closing remarks and future outlook

All the above-mentioned findings suggest the importance of *A. rugosa* for East Asian traditional medicine, which can be expected to extend to other regions, similar to the already more popular herbs from *Lamiaceae*. Two American species—currently more common as ornamentals—should also be considered as prospective phytomedicines. For this to occur, more research is needed, both on whole crude drugs and on purified fractions and individual compounds.

The role of individual components in the determination of medicinal properties is yet to be elucidated and the mechanisms of action verified. This also applies to multi-herb formulas (most commonly administered in traditional medicine) in order to understand the contribution of each constituent as well as any potentially synergistic or additive relationships.

Another question concerns how genetic diversity, already existing in the form of many ornamental varieties, affects phytochemical composition and medicinal properties, as exemplified by the case of *A. mexicana* subspecies (Estrada-Reyes et al. 2004).

Issues relating to the diversity and variability of essential oil composition and yield must be resolved as a prerequisite for proper quality management and standardization procedures. In this respect, the biotechnological approach may become the appropriate solution. It applies equally to the composition of non-volatile phytochemicals, such as tilianin and other flavones, phenolic acids, rare diterpenoids and triterpenoids. The latter two groups—except for ursolic acid—have not been sufficiently studied in terms of their bioactivity or the possibility of obtaining applicable amounts for pharmaceutical use. These compounds are also promising as biological pesticides, especially in the protection of stored crops attacked by pest invertebrates.

In the near future, we will most likely witness a flurry of publications dealing with various aspects of bioactivity and molecular phytochemistry in *Agastache*. We hope that this review will inspire more researchers to consider those beautiful and healthy plants worth studying, as well as funding agencies in supporting such research.
